# FBG-Based Multi-Parameter Sensor for Harsh Transformer Conditions: Decoupling Packaging for Simultaneous Temperature, Pressure, and Moisture Measurement

**DOI:** 10.3390/s26134243

**Published:** 2026-07-04

**Authors:** Debao Wang, Shangang Ma, Fubao Jin, Ruiming Wang

**Affiliations:** School of Energy and Electrical Engineering, Qinghai University, Xining 810016, China; wdb183103430@163.com (D.W.); jinfubao@163.com (F.J.); 13545467532@163.com (R.W.)

**Keywords:** transformer monitoring, fiber bragg grating, integrated fiber-optic sensor, sensor structure design, decoupling packaging

## Abstract

The oil-immersed environment within power transformers is characterized by high temperatures, strong electric fields, and severe electromagnetic interference, posing significant challenges for simultaneous multi-parameter monitoring. Conventional electrical sensors are susceptible to electromagnetic interference, whereas typical integrated fiber Bragg grating (FBG) sensors exhibit cross-sensitivity and reliability issues under such harsh operating conditions. To address these challenges, this paper proposes an integrated FBG-based sensor. Through specialized material and structural design, each sensing element is engineered to respond predominantly to its target parameter at the physical level. This approach effectively mitigates cross-sensitivity, enabling high-precision simultaneous measurement of oil temperature, pressure, and moisture content. Under simulated transformer oil conditions, the sensor achieved a temperature sensitivity of 17.1 pm/°C, a pressure sensitivity of approximately 4 nm/MPa, and a moisture sensitivity of 7.775 × 10^−4^ nm/%RS (equivalent to 6.37 × 10^−4^ nm/ppm at 40 °C). The results also confirmed excellent linearity, repeatability, and resistance to cross-sensitivity. These findings demonstrate that the proposed integrated FBG sensor can achieve stable multi-parameter measurement and effective decoupling under the tested transformer-oil conditions, indicating its potential for engineering application in transformer online monitoring.

## 1. Introduction

As a critical component of the power system, the safe and stable operation of power transformers is fundamental to ensuring reliable electricity transmission [1]. Improving the accuracy, real-time performance, and comprehensiveness of transformer condition monitoring has become essential for advancing intelligent operation and maintenance of power equipment [2]. Key parameters of transformer oil—such as temperature, moisture content, and internal pressure—are closely related to the insulation condition, thermal performance, and fault development of the transformer. Therefore, long-term and accurate measurement of these parameters is of great significance for effective condition assessment and fault detection in transformers. Conventional electrical sensors are susceptible to interference in strong electromagnetic environments and often face limitations in data acquisition, signal transmission, and on-site power supply [3]. In contrast, fiber Bragg grating (FBG) sensors, as optical sensing devices, offer high sensitivity, ease of miniaturization, immunity to electromagnetic interference, and excellent corrosion resistance [4]. These advantages make them particularly suitable for condition monitoring within the complex internal environment of transformers.

In recent years, FBG sensors have become a research focus in the sensing field and have been widely used for single-parameter measurements of temperature, strain, pressure, magnetic field, and humidity [5,6,7,8,9]. However, with the increasing demands of equipment condition monitoring, the development of multifunctional FBG sensors capable of simultaneously detecting multiple parameters has emerged as a key challenge [10]. To address this, extensive research has been conducted worldwide. For instance, Jun et al. proposed a multi-parameter FBG sensor for industrial hydraulic pipelines, which showed good agreement with electrical and piezoelectric sensors [11]. Wang et al. developed a hybrid long-period fiber grating by combining multimode and single-mode fibers for simultaneous strain and temperature measurement [12]. Flavio et al. designed an FBG sensing system capable of concurrently monitoring the surrounding refractive index, strain, and temperature [13]. Wang et al. employed fiber multiplexing technology to interconnect multiple sensors, achieving multi-parameter monitoring in industrial processes [14]. However, the aforementioned sensor designs did not fully account for applications in complex and harsh environments. In the specific context of power transformers—characterized by severe operating conditions and complex physical fields—achieving integrated multi-parameter FBG sensing presents critical challenges. These include cross-sensitivity among measured parameters and the difficulty of designing packaging structures compatible with such demanding environments [15].

To cope with the harsh operating conditions inside a power transformer, this paper proposes an integrated FBG sensor adopting a layered isolation architecture for simultaneous measurement of temperature, pressure, and moisture. The temperature sensing unit employs a ceramic tube with point-contact; the pressure sensing unit introduces a gap to achieve efficient pressure transfer; the moisture sensing unit utilizes a polyimide coating together with hardware-reuse temperature compensation to realize temperature–moisture decoupling; and the non-metallic package ensures electrical insulation. Experimental results show that the temperature sensitivity (17.1 pm/°C) and pressure sensitivity (4 nm/MPa) are comparable to values reported in the literature [11,12,13], while the moisture sensitivity (0.7775 pm/%RS) is slightly lower. Importantly, the pressure-to-temperature cross-sensitivity is only 0.09 pm/kPa, which is lower than the reported 1 pm/kPa [15], and the residual pressure drift after compensation is 0.3 kPa, outperforming the conventional >1 kPa/°C. In addition, the temperature–moisture cross-interference is effectively suppressed by reusing the temperature FBG for compensation. In summary, under the specific operating environment of power transformers, the proposed sensor maintains adequate primary sensitivities while significantly reducing cross-sensitivities, thereby achieving highly integrated multi-parameter decoupled measurement. These results provide a promising integrated sensing solution for online monitoring of temperature, pressure, and moisture content in transformer oil.

## 2. Design of the Integrated Fiber Bragg Grating Sensor

### 2.1. Temperature Sensing Design

**Figure 1:**
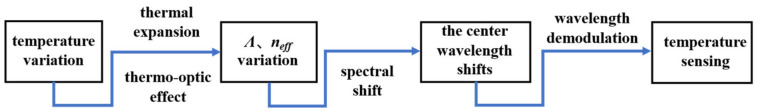
Schematic diagram of fiber grating temperature sensing.

The Bragg wavelength of an FBG shifts with temperature and strain, as described by Equation (1) [16,17]. Its measurement principle is illustrated in Figure 1.

(1) $$\frac{\Delta \lambda }{\lambda }=(1-P_{e})\varepsilon +(\alpha +\xi )\Delta T$$

In this expression, Δ*λ* represents the Bragg wavelength shift, *λ* is the initial (unperturbed) central wavelength, *P_e_* is the effective photo-elastic coefficient of the optical fiber, *ε* is the axial strain experienced by the optical fiber, *α* is the thermal expansion coefficient of the fiber, *ξ* is the thermo-optic coefficient, and Δ*T* denotes the temperature change.

However, because the Bragg wavelength shift of an FBG is inherently sensitive to both strain and temperature, practical temperature measurements are often susceptible to strain-induced cross-interference [18]. To mitigate this cross-sensitivity under combined temperature and pressure effects, this paper proposes a strain-isolated temperature sensing structure based on high-modulus ceramic packaging. The temperature sensing element is encapsulated within 99% alumina (Al_2_O_3_) ceramic. The high thermal conductivity of alumina is beneficial for heat transfer to the FBG and is expected to improve thermal response. At the same time, the ceramic material has a high elastic modulus (approximately 300–400 GPa), forming a rigid shielding shell that effectively resists external mechanical stress.

**Figure 2:**
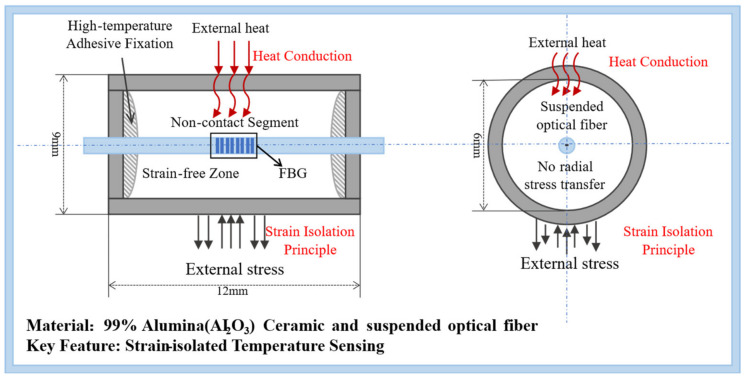
Structural diagram of the FBG temperature sensor.

The structure adopts a point-contact fixation method: the FBG is axially arranged within a ceramic tube and fixed only at its two axial endpoints using a high-temperature-resistant adhesive, leaving the majority of the intermediate fiber segment in a non-contact state with the inner wall. Consequently, external loads and most transverse stresses are primarily borne by the high-rigidity ceramic tube, while the internal grating responds exclusively to temperature field variations. A schematic diagram of the proposed FBG temperature sensing unit is presented in Figure 2.

It is important to note that the alumina ceramic tube not only serves as a rigid mechanical shield but also contributes to the temperature response of the packaged FBG through its own thermal expansion. The thermal expansion coefficient of 99% alumina ceramic is considerably larger than that of silica fiber. As temperature increases, the ceramic tube expands longitudinally, and this expansion is transferred to the grating via the adhesive fixation points at the two ends, superimposing an additional tensile thermal strain onto the intrinsic thermal response of the fiber. As a result, the measured temperature sensitivity of the packaged FBG is higher than that of a bare fiber, as observed in subsequent tests. This sensitivity enhancement is beneficial for practical measurement, as it improves the temperature response sensitivity without compromising the strain-isolation function. Overall, this design ensures strong immunity to cross-sensitivity.

### 2.2. Pressure Sensing Design

**Figure 3:**
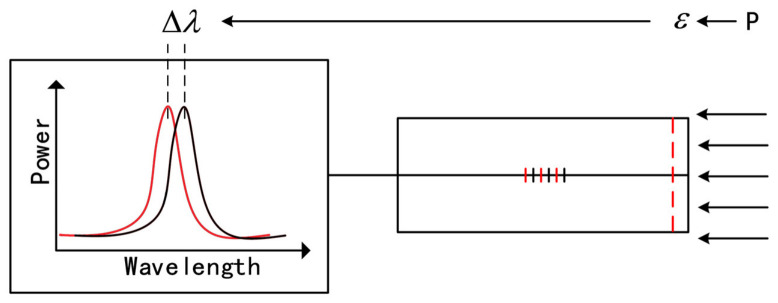
Principle of FBG pressure sensing.

Figure 3 shows the wavelength demodulation principle of the FBG pressure sensor. External pressure P induces fiber strain and Bragg wavelength shift Δλ; pressure is measured via the quantitative relationship between Δλ and P, as in Equation (1). Due to the inherently low strain sensitivity of bare optical fibers, they are unsuitable for practical measurements. Polymer packaging is commonly employed to effectively enhance the sensitivity of FBG pressure sensors [18]. However, polymer-packaged pressure sensors immersed in fluids are typically subjected to pressure from all directions. To enable unidirectional pressure measurement and prevent interference from extraneous pressure on polymer deformation, researchers have introduced a rigid outer shell around the polymer to shield against stresses from other orientations. This configuration is known as the traditional pressure-sensitive tank-type sensor [19]. In this work, the polymer sensitizing element is made of fluorosilicone rubber. When external pressure acts on its end face, the rubber column undergoes axial compression, with the strain direction opposite to the pressure direction (i.e., the compressive strain is negative). According to the FBG strain sensing principle, a negative strain causes the Bragg wavelength to shift toward shorter wavelengths (blue shift); therefore, the wavelength decreases as pressure increases. This relationship is fully consistent with the subsequent experimental results.

However, in the practical environment of a transformer, prolonged exposure to high-temperature oil introduces new challenges for this sensing structure. First, the damping effect between the polymer and the rigid shell reduces the strain response of the polymer elastomer when subjected to external axial stress. Second, the rigid shell constrains the free deformation of the polymer under external stress, indirectly affecting the axial strain transferred to the FBG. Furthermore, when the oil temperature rises, thermal expansion of both the polymer and the rigid shell generates significant friction between them, which impedes the axial strain of the polymer and consequently reduces measurement sensitivity.

**Figure 4:**
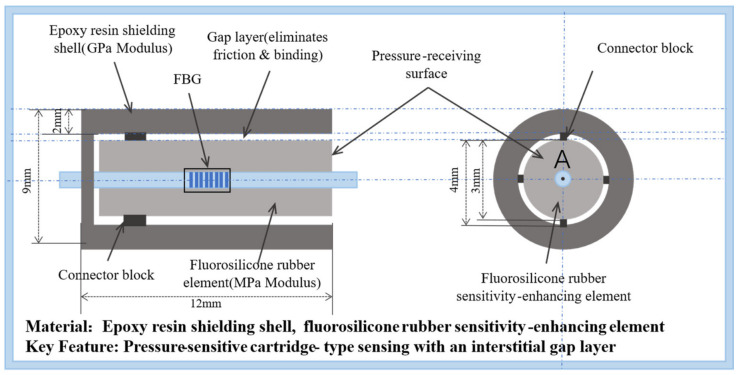
Structural diagram of the FBG pressure sensor.

To address these limitations, this paper proposes an improved design based on the conventional structure, a half-sectional view of which is presented in Figure 4. An epoxy resin shielding shell with an elastic modulus on the order of GPa is introduced around the internal fluorosilicone rubber sensitivity-enhancing element, and a specific gap (void layer) is reserved between them. The GPa-level stiffness is substantially higher than that of the fluorosilicone rubber (MPa-level). This configuration retains the advantages of the sensitivity-enhancing tank, with only Area A in Figure 4 serving as the force-bearing surface for pressure sensing, thereby ensuring measurement directionality. At the same time, the reserved gap is designed to reduce friction and deformation constraints between the sensitive element and the outer shell, thereby enabling the external pressure signal to be converted into axial strain on the grating with high efficiency. In addition, epoxy resin has been widely used in high-voltage electrical equipment and can provide electrical insulation compatibility between the sensor and the transformer.

### 2.3. Moisture Sensing Design

**Figure 5:**
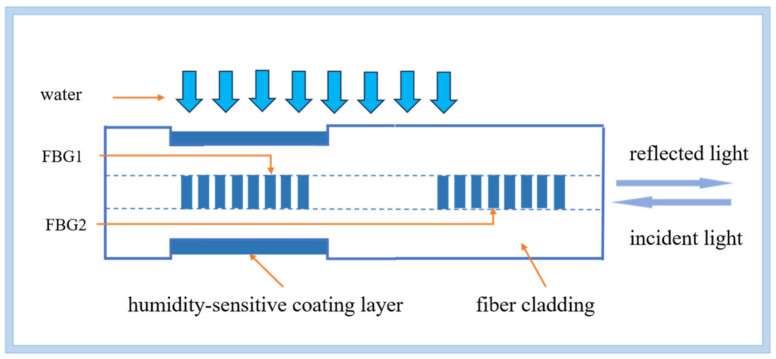
Schematic of the FBG moisture sensor.

In general, FBGs exhibit low intrinsic sensitivity to humidity variations. Therefore, for humidity detection, a hygroscopic (humidity-sensitive) coating is typically applied to the fiber surface; the moisture content is measured indirectly via the strain induced by the coating’s swelling upon water absorption [20]. Temperature also affects the wavelength through the thermo-optic and thermal expansion effects, thus requiring compensation [21]. To compensate for the influence of temperature on moisture measurement, one approach is to adopt a dual-FBG humidity sensor configuration, as illustrated in Figure 5. In this configuration, FBG1 is coated with a hygroscopic polyimide film and is therefore sensitive to both temperature and humidity, whereas FBG2 is sensitive only to temperature. By comparing the wavelength shifts of the two gratings, the humidity-induced component can be isolated, from which the moisture content is then derived. Building upon this approach and to make efficient use of the limited interrogator bandwidth while simplifying the probe structure, this work further adopts an integrated design: rather than incorporating a separate dedicated reference grating, the strain-isolated temperature sensing unit described in Section 2.1 is employed as the temperature reference, and both have the same fiber geometry. This design reuses the existing temperature sensor for compensation, eliminates the need for a redundant grating, reduces the overall probe dimensions, and conserves interrogator bandwidth.

In fabricating the FBG humidity sensing unit, a femtosecond laser is used to etch and thin the fiber cladding, reducing the cladding diameter from the standard 125 μm to approximately 80 μm, thereby enhancing the strain transfer efficiency induced by the subsequent moisture-sensitive coating. After etching, the fiber is rinsed with deionized water and dried. The cleaned grating region is then immersed in a silane solution for 1 min, removed, and dried. Following this surface pretreatment, a polyimide film with a thickness of approximately 0.1 mm is deposited onto the grating region via dip-coating. After the dip-coating process, a two-step thermal curing protocol is applied to achieve full imidization of the polyimide film and to form strong adhesion with the fiber substrate. Subsequently, the coated fiber section was encapsulated within a porous protective layer to enhance its mechanical protection. The humidity FBG has a grating length of 5 mm. The etched length (i.e., the cladding-reduced region) is approximately 8 mm, covering the entire grating and extending about 3 mm beyond it. The polyimide coating length is 8 mm, fully covering the grating, with a gradual transition zone of about 1 mm at each end to minimize stress concentration at the coating edges.

### 2.4. Design of the Integrated Temperature, Pressure, and Moisture Sensor

#### 2.4.1. Fundamental Design Principles

To interrogate the three FBGs simultaneously through a single optical fiber, we employ wavelength division multiplexing (WDM) [22]. Specifically, the three FBGs were assigned center wavelengths of 1540, 1550, and 1560 nm, with a channel spacing of 10 nm. This allocation was determined through a spectral overlap analysis. Under full-scale operation, the maximum wavelength excursions of the pressure grating (0–1 MPa), temperature grating (20–120 °C), and humidity grating (0–100% RS) are approximately 4.0 nm, 1.71 nm, and 0.08 nm, respectively. Accounting for a fabrication tolerance of ±0.2 nm and a minimum guard band of 2 nm between adjacent channels, the required minimum separations between the pressure–temperature and temperature–humidity channel pairs are approximately 5.3 nm and 3.3 nm, respectively. The allocated 10 nm spacing satisfies these requirements for both channel pairs with a margin exceeding 4 nm, while remaining fully within the interrogator’s 1527–1568 nm spectral window. The specifications of the optical fibers are listed in Table 1.

#### 2.4.2. Structural Design

The integrated sensor adopts an outward-to-inward layered spatial configuration, with each layer performing a distinct isolation function. The humidity sensing unit is positioned at the outermost layer and is encased in a perforated support sleeve, which allows the transformer oil to circulate freely while protecting the grating from direct oil flow impingement. The polyimide-coated grating is in direct contact with the transformer oil, enabling prompt detection of moisture in the oil. Immediately inward, a polytrifluorochloroethylene (PCTFE) film with a thickness of 0.2 mm serves as the moisture barrier. PCTFE exhibits extremely low moisture permeability, negligible thermal resistance, and high elasticity, enabling effective blockage of moisture ingress without compromising temperature or pressure transmission, thereby protecting the pressure and temperature sensing units from humidity interference. Inside the moisture barrier is the temperature sensing unit, encapsulated in an alumina ceramic tube and responsive solely to temperature variations. Further inward, a thermal insulation layer fabricated from a high-temperature-resistant thermoplastic material is installed. This layer features an axial opening on its side wall, which is sealed with a corrugated diaphragm. External transformer oil pressure acts on the diaphragm, and the resulting diaphragm deflection is transmitted to the fluorosilicone rubber elastomer, achieving high-sensitivity pressure transfer while simultaneously attenuating thermal interference. The pressure sensing unit, consisting of a fluorosilicone rubber elastomer and an epoxy resin shielding shell, occupies the innermost position.

**Figure 6:**
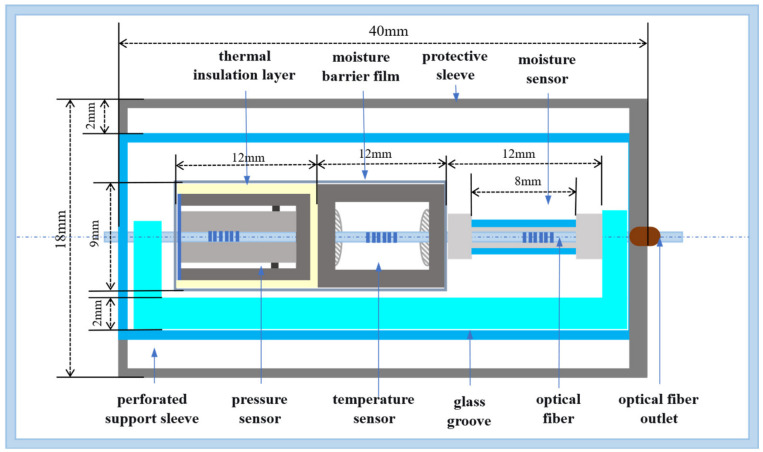
Structural diagram of the integrated FBG sensor.

**Figure 7:**
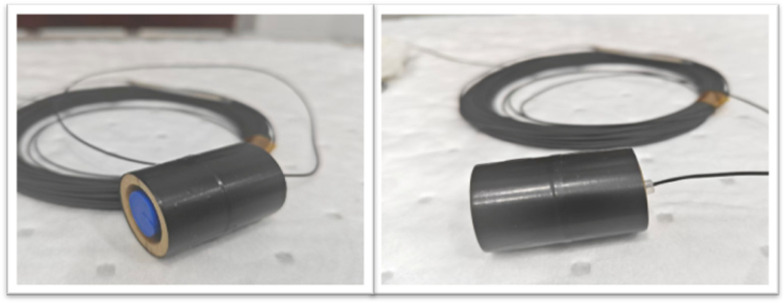
Physical photograph of the integrated FBG sensor.

With regard to overall packaging, a borosilicate glass groove serves as both the rigid support substrate for the entire sensing assembly and the fiber fixing platform. The optical fiber is precisely aligned and bonded within the V-shaped positioning groove of the glass substrate using a high-temperature-resistant epoxy adhesive. The thermal expansion coefficient of borosilicate glass is highly matched to that of the silica optical fiber, which fundamentally eliminates thermally induced mismatch stress and avoids spurious wavelength shifts. The polyimide-coated humidity-sensitive grating is located at the outermost end of the glass groove, directly exposed to the oil flow channel of the perforated support sleeve; the temperature and pressure gratings are sequentially arranged in the inner segmented regions of the glass groove. The moisture barrier film isolates the pressure and temperature gratings from the humidity grating. Both the thermoplastic thermal insulation layer and the epoxy resin shielding shell are mounted using the glass groove as the installation reference, thereby further partitioning the pressure grating from the temperature grating. The glass groove is fixed as a whole inside the perforated sleeve, providing unified mechanical support for all internal components and isolating them from oil flow impact and mechanical vibration within the transformer. The lead fibers from each grating are routed out through dedicated exit ports in the sleeve, and protective sheaths are installed at the exit ports to safeguard the fibers from tension, abrasion, and vibration damage. An outermost protective sleeve encloses the entire assembly, offering reliable mechanical protection and ensuring that the internal gratings remain unaffected by external stresses that could perturb the measurement signals. Figure 6 and Figure 7 present the structural schematic diagram and the physical photograph of the integrated sensor, respectively.

This spatial configuration enables the three sensing units to operate cooperatively in an integrated manner while providing effective physical isolation among pressure, temperature, and moisture measurements, thereby mitigating the influence of cross-interference. Furthermore, borosilicate glass possesses excellent electrical insulation properties and superior compatibility with transformer oil—exhibiting no swelling and no leachable substances during long-term immersion. It securely anchors the FBG probe and effectively isolates the gratings from the complex external environment, further enhancing the long-term operational stability of the sensor. The fully non-metallic housing also ensures the insulation safety of the sensor when operating in the strong electromagnetic field environment inside power transformers.

## 3. Experimental Results and Analysis

### 3.1. Experimental Setup

**Figure 8:**
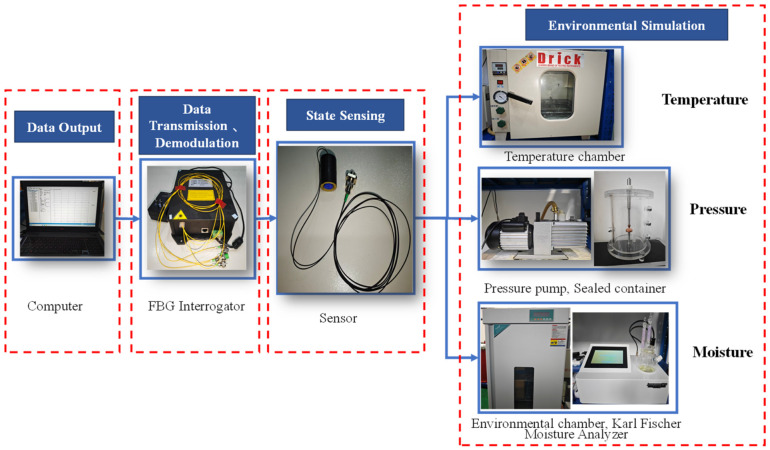
Experimental setup.

To evaluate the sensing performance of the proposed sensor, an experimental platform was established, as illustrated in Figure 8. A temperature-controlled oven (Shandong Derrick Instrument Co., Ltd., Jinan, China) was employed to assess temperature measurement performance. The pressure testing platform consisted of a pressure pump (Shanghai Shuang’e Refrigeration Equipment Co., Ltd., Shanghai, China) combined with a sealed vessel (Linquan County Chengpin Machinery Processing Factory, Fuyang, China). Moisture testing was conducted using a temperature and moisture chamber (Hebi Jingzhong Technology Co., Ltd., Hebi, China). The measured data were transmitted from the demodulation module to the host computer software (QSfiberUI (version v1.1.0.0)) for acquisition and processing.

During the experiments, a fiber Bragg grating interrogator (Model: VG-FBGM104A) (Tianjin Qiushi Feibo Technology Co., Ltd., Tianjin, China) was used for wavelength demodulation. The interrogator employs a tunable semiconductor laser as the light source, and its main technical specifications are listed in Table 2. It should be noted that the 40 pm scanning step represents the spectral sampling interval of the tunable laser scan, rather than the final wavelength resolution or absolute accuracy of the demodulated FBG peak. The Bragg wavelength is determined by applying a built-in Gaussian peak-fitting algorithm to multiple discrete spectral sampling points around the reflection peak; therefore, the peak center can be estimated with sub-sampling precision. In this study, the value of ±0.3 pm is interpreted as the wavelength repeatability, whereas the absolute wavelength accuracy is ±1 pm, as specified in Table 2.

### 3.2. Experimental Analysis

#### 3.2.1. Temperature Sensing Performance

To simulate actual transformer oil temperature monitoring conditions, the sensor was immersed in transformer oil and placed within a temperature-controlled oven. During the experiment, the temperature was increased from 20 °C to 140 °C in increments of 10 °C. At each temperature setpoint, the oven temperature was maintained for 30 min to ensure complete thermal stabilization of the oil sample. The central wavelength of the temperature sensing unit was then recorded, and this procedure was repeated three times. In the data processing procedure, to suppress random noise interference, the discrete wavelength data obtained from multiple sets of experiments were first arithmetically averaged. Meanwhile, the standard deviation at each temperature point was calculated based on the repeated test data, and the measurement results at different temperatures are presented as discrete data points with error bars (±1 SD) to reflect the degree of data dispersion and the repeatability of the measurements. Subsequently, linear regression fitting was performed with the mean wavelength and temperature as variables to establish the quantitative relationship between the two.

**Figure 9:**
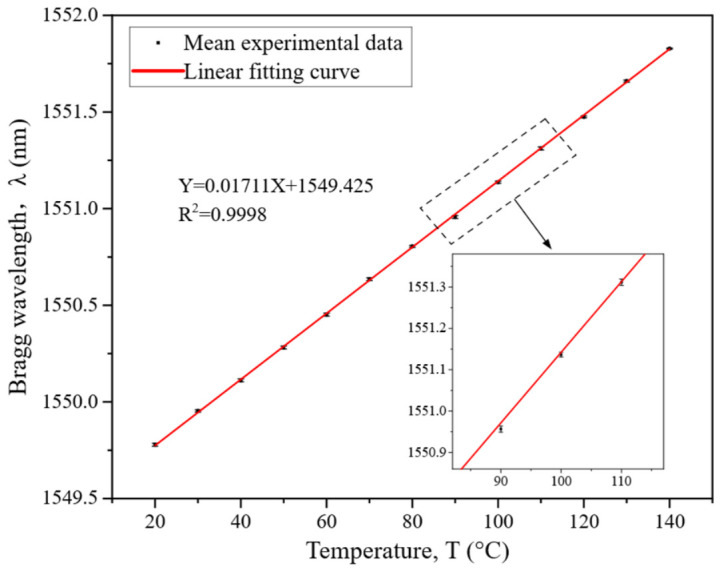
Bragg wavelength versus temperature for the temperature sensing unit immersed in transformer oil. Data points represent the mean of three repeated thermal cycles, with error bars indicating ±1 standard deviation. Most error bars are smaller than the symbol size. The solid line is the linear fit (R^2^ = 0.9998). Inset: enlarged view of the 85–115 °C region showing error bars below 8 pm.

The fitting results are shown in Figure 9. Based on three repeated experiments, the mean standard deviation is 6.33 pm, the maximum standard deviation is 7.64 pm, and the root-mean-square error relative to the fitted line is 8.37 pm, corresponding to an equivalent temperature error of 0.49 °C, measurement accuracy of 0.5 °C. Linear regression of the experimental data yields a correlation coefficient (R^2^) of 0.9998. Furthermore, the temperature sensitivity is calculated to be 17.1 pm/°C. These indicators demonstrate that the temperature sensing unit exhibits excellent repeatability and linearity over the full range, and its detection accuracy is superior to the conventional accuracy range (0.5–1 °C) of built-in temperature sensors in traditional transformers.

**Figure 10:**
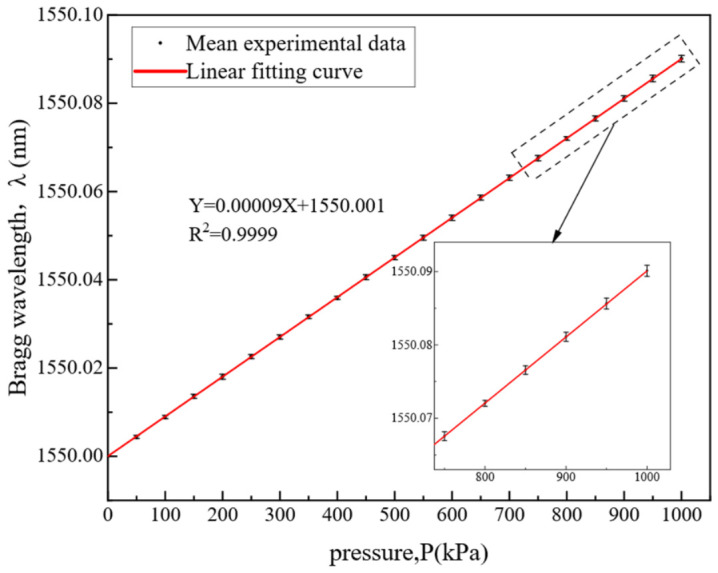
Relationship between the central wavelength of the temperature fiber and pressure.

Although an isolation design has been implemented, under external pressure, the ceramic tube itself undergoes slight compression, and the base or housing experiences minor bending. Meanwhile, the adhesive used to fix the optical fiber has a certain elasticity, which can transmit part of the pressure. As a result, the temperature measurement may also be affected. Therefore, a cross-sensitivity test was conducted. The sensor was placed in a sealed chamber under constant temperature conditions. Pressure was adjusted within the range of 0–1000 kPa (gauge pressure) using a pressure pump, and the central wavelength shift was recorded at 50 kPa intervals. Two measurement cycles were performed, with the results shown in Figure 10. Least-squares fitting indicates that the cross-sensitivity coefficient of the temperature FBG to pressure is approximately 0.09 pm/kPa. These results demonstrate that the pressure-induced interference in temperature measurement is negligible in engineering practice after applying the proposed compensation method under normal operating conditions of power transformers.

#### 3.2.2. Pressure Sensing Performance Evaluation

**Figure 11:**
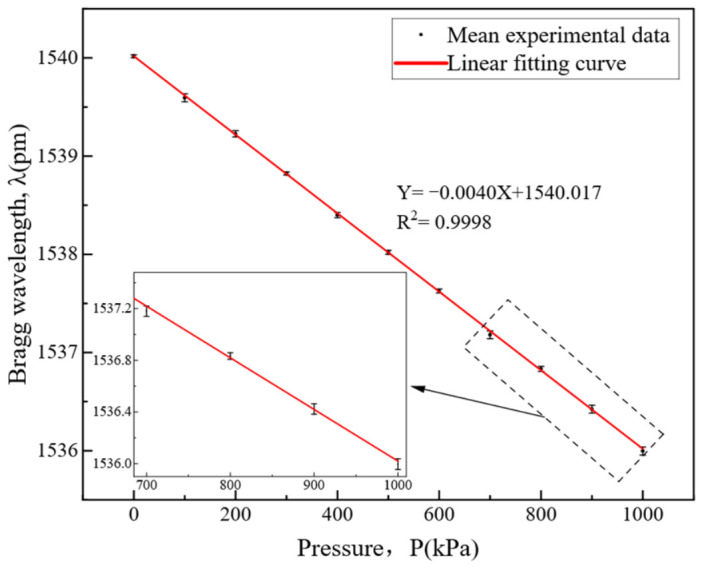
Wavelength versus pressure for the pressure sensing unit. Data points show the mean of three repeated experiments, with error bars of ±1 standard deviation. Solid line: linear fit (R^2^ = 0.9998, sensitivity = 4 nm/MPa). Inset: 700–1000 kPa enlarged view confirming excellent repeatability.

To evaluate the pressure-sensing performance of the proposed sensor, experiments were conducted on a pressure testing platform under constant temperature conditions. During the experiments, pressure was varied from 0 to 1 MPa (gauge pressure) in increments of 100 kPa, with measurement data recorded at each step. Three repeated experiments were performed. The discrete wavelength data obtained from these experiments were arithmetically averaged. Subsequently, linear regression fitting was applied to the averaged wavelength values against pressure, yielding the central wavelength-pressure relationship curve shown in Figure 11. The experimental results give a correlation coefficient (R^2^) of 0.9998, indicating excellent output linearity. From three repeated experiments, the mean standard deviation is 28.87 pm, the maximum standard deviation is 41.63 pm, and the root-mean-square error relative to the fitted line is 17.0 pm, corresponding to an equivalent pressure error of 4.25 kPa, measurement accuracy of 5 kPa. This error level is much smaller than the pressure changes (tens to hundreds of kPa) that may occur during transformer faults. These indicators demonstrate that the pressure sensor possesses high measurement accuracy and repeatability.

**Figure 12:**
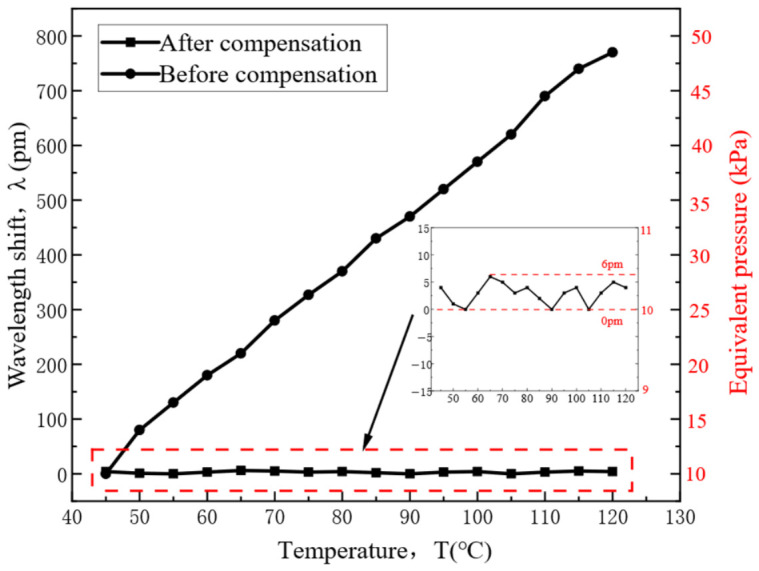
Equivalent pressure shift before and after temperature compensation.

Since pressure measurements are often susceptible to temperature variations, experiments were conducted to characterize the temperature-dependent measurement stability of the proposed pressure sensor, thereby validating the effectiveness of the proposed temperature compensation scheme in suppressing temperature interference. Figure 12 presents the test results within the temperature range of 45–120 °C at a pressure of 10 kPa, both without temperature compensation and after applying the compensation method described in Section 2. As illustrated in the figure, the central wavelength of the sensor increased from 1540.21 nm to 1540.970 nm as the temperature rose, corresponding to a total wavelength drift of approximately 760 pm. As shown in Figure 12, this drift is equivalent to an applied pressure of 49.27 kPa, representing a substantial measurement error. After introducing the temperature compensation grating, the wavelength drift within the 45–120 °C range was controlled to within 6 pm, with an average value of approximately 3.06 pm. In other words, for the pressure sensor with temperature compensation, the residual wavelength drift induced by temperature variations corresponds to an equivalent pressure of only approximately 0.3 kPa. Compared to the uncompensated case, the measurement error is significantly reduced. Relative to the pressure variations ranging from tens to hundreds of kilopascals that may occur during transformer fault conditions, this error level is negligible. Therefore, the adopted temperature compensation strategy can be considered effective in suppressing temperature-induced interference.

#### 3.2.3. Moisture Sensing Performance Evaluation

Given the difficulty of precisely controlling the absolute water content of transformer oil under current laboratory conditions, an indirect gas–liquid equilibrium method was adopted for humidity calibration. The transformer oil used was a commercial naphthenic mineral insulating oil (Type: KI25X) (Wuhan KanoS Technology Co., Ltd., Wuhan, China). Prior to calibration, 2 L of oil was dehydrated in a vacuum oven at 80 °C for 48 h. The initial moisture content after dehydration was verified to be below 5 ppm using a Karl Fischer moisture titrator (Model: JZ WS 9000) (Hebi Jingzhong Technology Co., Ltd., Hebi, China). The dehydrated oil sample was placed in an open glass beaker and transferred to a temperature- and humidity-controlled chamber, where the temperature was maintained at 40 ± 0.5 °C and the initial relative humidity was set to 35% RH. Based on the principle of gas–liquid phase equilibrium, the dried oil absorbs ambient moisture until the relative saturation of water in the oil (%RS) equals the relative humidity inside the chamber. To accelerate moisture diffusion, the oil sample was continuously stirred at 200 rpm using a magnetic stirrer. An equilibration time of 8 h was adopted for each humidity calibration point. This duration was determined based on the most severe condition (95% RH), under which the gas–liquid mass transfer resistance is the highest and equilibration is the slowest. Actual monitoring showed that the change rate of moisture content in the oil stabilized within ±1 ppm/h after approximately 7 h. Therefore, the 8 h holding time ensures complete equilibration across the entire humidity range (35–95% RH).

**Figure 13:**
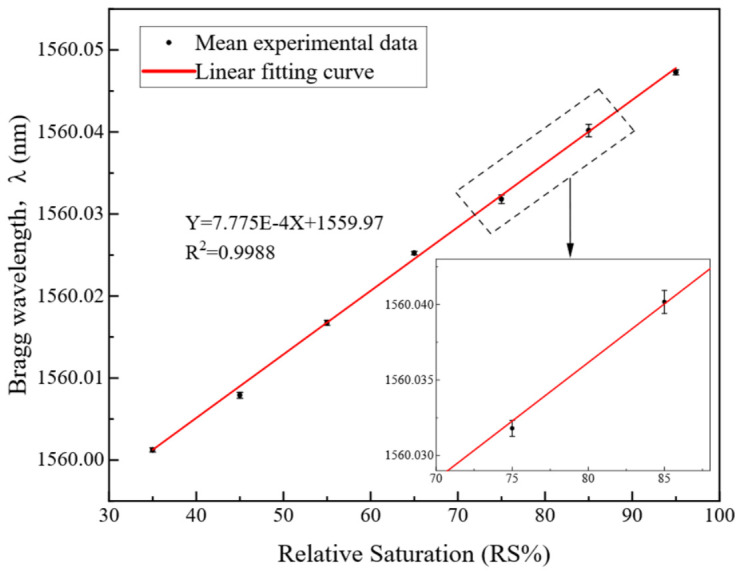
Bragg wavelength versus relative saturation (RS%) for the moisture-sensitive unit. Data points represent the mean of three repeated experiments, with error bars of ±1 standard deviation. Solid line: linear fit (R^2^ = 0.9988, sensitivity = 7.775 × 10^−4^ nm/%RS). Inset: magnified 70–85% RS range confirming excellent repeatability.

Under these conditions, the chamber humidity was increased from 35% RH to 95% RH in increments of 10% RH. Meanwhile, the absolute water content at each calibration point was independently verified using a Karl Fischer moisture titrator, and the FBG sensor wavelength was recorded after confirming that the moisture content had stabilized. This experiment was repeated three times. Linear regression fitting was performed on the collected data, yielding the relationship between the central wavelength and humidity shown in Figure 13. The experimental results give a correlation coefficient (R^2^) of 0.9988, indicating good output linearity. Based on three repeated experiments, the mean standard deviation is 0.389 pm, the maximum standard deviation is 0.764 pm, and the root-mean-square error relative to the fitted line is 3.83 pm. In addition, the sensitivity of the moisture sensing probe is calculated to be 7.775 × 10^−4^ nm/% RS. These indicators demonstrate that the proposed sensor offers high measurement accuracy and operational stability.

To convert relative saturation to absolute moisture content in transformer oil (expressed in ppm), the actual moisture content *W* (ppm) is calculated using the following fundamental Equations (2) and (3) [23]:

(2) $$\log_{10}S=A-\frac{B}{T_{K}}$$

(3) $$W=\frac{RS\%}{100}S$$

In these equations, *S* represents the saturation solubility of moisture in the oil at the given temperature (in ppm), and *T_K_* denotes the thermodynamic temperature (*T_K_* = 273.15 + *T*). The coefficients *A* and *B* are characteristic constants of the oil, taken as 7.0895 and 1567, respectively. Based on this conversion, the sensitivity of the moisture sensor is determined to be 6.37 × 10^−4^ nm/ppm at 40 °C.

**Figure 14:**
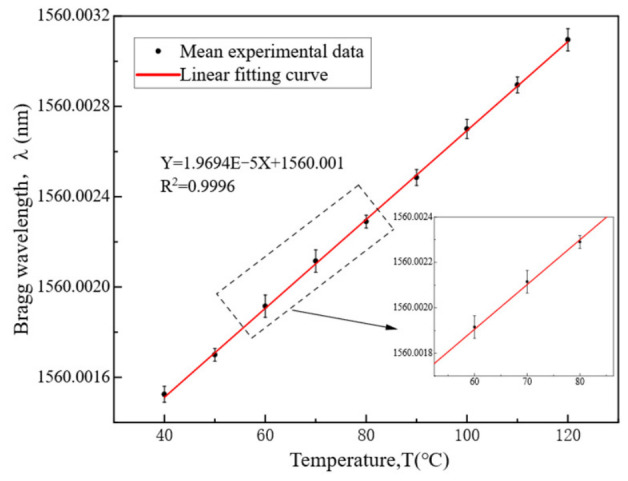
Central wavelength of the moisture-sensing FBG versus temperature.

To verify the effectiveness of the moisture detection compensation scheme described in Section 2.3, a cross-sensitivity test of the humidity sensing unit was conducted. The sensor was placed in a temperature- and humidity-controlled chamber. The relative saturation was kept constant at 35% RS, while the temperature was cycled from 40 °C to 120 °C in steps of 10 °C, and the wavelength of the humidity FBG was recorded. The results are shown in Figure 14. Least-squares fitting indicates that the residual cross-sensitivity of the compensated humidity FBG to temperature is approximately 0.0197 pm/°C. This result demonstrates that, compared with the humidity sensitivity of 0.7775 pm/%RS, the proposed compensation scheme greatly reduces temperature interference. Under normal operating conditions of an actual power transformer, the interference of temperature on moisture measurement can be considered negligible. Therefore, the adopted design strategy can be regarded as effective in suppressing temperature-induced interference.

#### 3.2.4. Online Stability and Decoupling Verification

**Figure 15:**
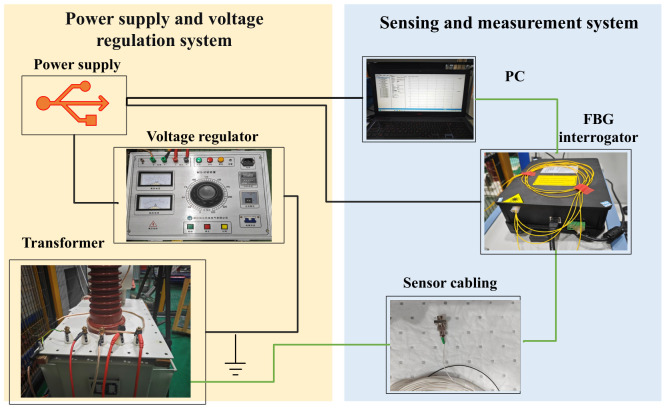
Transformer experimental setup.

The above experiments verified the fundamental response characteristics of the proposed sensor for each target parameter under controlled conditions, including sensitivity and linearity. However, in the actual operating environment of a power transformer, temperature, pressure, and humidity may vary simultaneously due to the coupled effects of thermal, electrical, and fluid multi-physical fields. Under such coupled conditions, whether each sensing unit can maintain a stable response to its target parameter with interference from non-target parameters is a critical factor determining the engineering applicability of the sensor. Therefore, a 30-day online monitoring test was conducted to evaluate the multi-parameter synchronous measurement performance, cross-sensitivity suppression capability, and operational stability of the sensor under actual transformer operating conditions. As shown in Figure 15, the experimental platform consists of a power transformer (Wuhan Guodian Xigao Electric Co., Ltd., Wuhan, China), voltage regulation equipment (Wuhan Guodian Xigao Electric Co., Ltd., Wuhan, China), a fiber Bragg grating interrogator (Tianjin Qiushi Feibo Technology Co., Ltd., Tianjin, China), and a host computer system (QSfiberUI (version v1.1.0.0)). To achieve multi-parameter decoupling, a 3 × 3 sensitivity matrix was constructed based on the calibrated sensitivity and cross-sensitivity coefficients of the three sensing channels.

**Figure 16:**
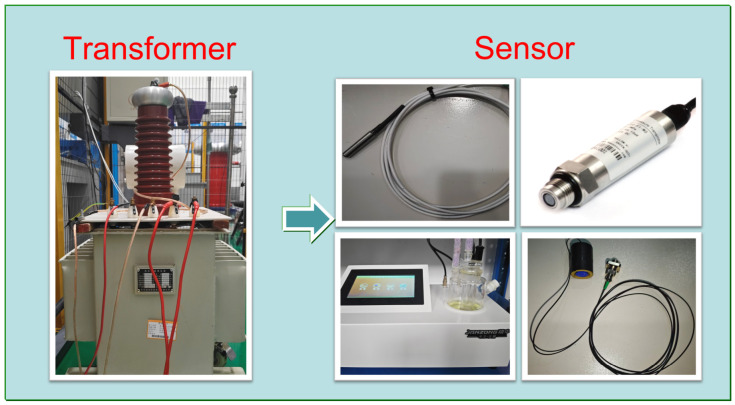
Diagram of experimental equipment.

To ensure comparability of the operation evaluation results, independent reference measurement devices were concurrently employed as controls. A high-precision platinum resistance thermometer (Model: MIK-WZP) (Sichuan Bodian Technology Co., Ltd., Mianyang, China)served as the temperature reference, a high-accuracy pressure sensor (Model: HM90-H2) (Nanjing Hongmu Technology Co., Ltd., Nanjing, China) served as the pressure reference, and a Karl Fischer moisture monitor (Hebi Jingzhong Technology Co., Ltd., Hebi, China) served as the moisture reference. These reference devices are illustrated in Figure 16.

**Figure 17:**
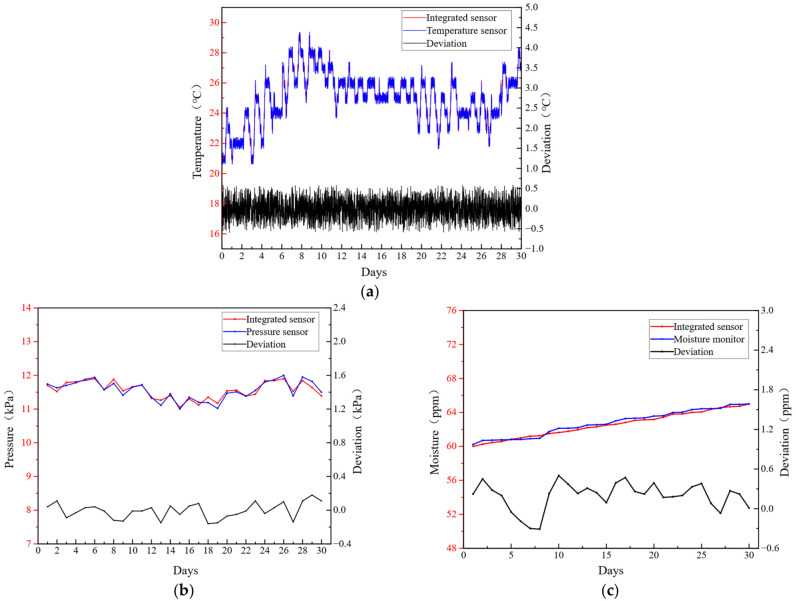
Long-term online monitoring results over the 30-day test. (**a**) Temperature, (**b**) Pressure, (**c**) Moisture content. In each subfigure, the upper curves show the FBG sensor readings and the reference instrument readings, while the lower curves present the corresponding difference curves.

During the testing period, measurement data were recorded at 15 min intervals. In the data processing stage, statistical analysis was performed on stable data segments within each sampling interval. Due to the minimal fluctuation and slow variation characteristics of pressure and moisture parameters, daily average values were employed to represent these parameters in order to clearly illustrate the macroscopic trends in the variation curves. The resulting parameter variations are presented in Figure 17.

Figure 17 presents the monitoring results of temperature, pressure, and moisture content obtained by the proposed sensor during 30 days of continuous operation. Throughout the test, the sensor maintained high-precision monitoring in the complex operating environment of the power transformer. The lower curves in Figure 17a–c show the deviations between the FBG sensor measurements and the reference instruments. The statistical results are as follows: root-mean-square deviations are 0.268 °C (temperature), 0.0914 kPa (pressure), and 0.281 ppm (moisture); maximum deviations are 0.58 °C, 0.18 kPa, and 0.50 ppm; mean absolute deviations are 0.211 °C, 0.0763 kPa, and 0.254 ppm; long-term drift rates are 0.0008 °C/day, 0.0005 kPa/day, and 0.002 ppm/day.

To quantitatively describe the residual coupling among the three sensing channels, a 3 × 3 sensitivity matrix was constructed using the calibrated primary sensitivities and residual cross-sensitivity coefficients. The relationship between the wavelength shifts and the parameter variations can be expressed as Equation (4).

(4) $$\left[\begin{array}{c} \Delta \lambda_{P}\\ \Delta \lambda_{T}\\ \Delta \lambda_{H} \end{array}\right]=\left[\begin{array}{ccc} S_{PP} & S_{PT} & S_{PH}\\ S_{TP} & S_{TT} & S_{TH}\\ S_{HP} & S_{HT} & S_{HH} \end{array}\right]\cdot \left[\begin{array}{c} \Delta P\\ \Delta T\\ \Delta H \end{array}\right]=\left[\begin{array}{ccc} -4 & 0.08 & 0\\ 0.09 & 17.1 & 0\\ 0 & 0.0197 & 0.7775 \end{array}\right]\cdot \left[\begin{array}{c} \Delta P\\ \Delta T\\ \Delta H \end{array}\right]$$

In this matrix, the wavelength shifts are expressed in pm, while ∆*P*, ∆*T*, and ∆*H* are expressed in kPa, °C, and %RS, respectively. Therefore, the coefficients in the pressure, temperature, and moisture columns have units of pm/kPa, pm/°C, and pm/%RS, respectively. The diagonal elements *S_TT_*, *S_PP_* and *S_HH_* were obtained from the pressure, temperature, and moisture calibration experiments, respectively. The off-diagonal elements *S_TP_*, *S_HT_* and *S_PT_* were obtained from the corresponding cross-sensitivity or compensation tests described above. In the practical inversion model, *S_TH_*, *S_PH_* and *S_HP_* were treated as negligible terms because their influence on the reconstructed parameters was limited within the tested range. The corresponding inverse matrix used for parameter reconstruction is given in Equation (5).

(5) $$\left[\begin{array}{c} \Delta P\\ \Delta T\\ \Delta H \end{array}\right]=\left[\begin{array}{ccc} -0.249974 & 0.001169 & 0\\ 0.001316 & 0.058473 & 0\\ -3.3335\times 10^{-5} & -0.001482 & 1.286174 \end{array}\right]\left[\begin{array}{c} \Delta \lambda_{P}\\ \Delta \lambda_{T}\\ \Delta \lambda_{H} \end{array}\right]$$

Using this inverse matrix, the measured wavelength shifts can be converted into the corresponding variations in pressure, temperature, and moisture content, thereby enabling matrix-based multi-parameter decoupling.

These results confirm that the integrated sensor not only exhibits excellent long-term stability and repeatability but also reliably maintains its initial calibration relationship in complex electromagnetic, temperature–humidity, and oil-immersed environments, thereby significantly reducing measurement errors caused by output drift. In summary, the 30-day online comparative test preliminarily demonstrated the operational stability and measurement consistency of the proposed sensor under the tested transformer operating conditions, revealing its engineering application prospects in power equipment condition monitoring.

## 4. Conclusions

To meet the demand for multi-parameter condition monitoring inside power transformers, this paper presents an integrated FBG sensor capable of simultaneously measuring oil temperature, internal pressure, and moisture content. Distinguished from conventional FBG sensors, the proposed sensor adopts a layered isolation architecture that achieves physical-level decoupling of the three sensing responses. Specifically, the temperature unit employs a point-contact suspension structure within a high-modulus alumina ceramic tube, physically blocking the transmission path of external strain. The pressure sensing unit forms a defined spatial gap between the fluorosilicone rubber sensitivity-enhancing element and the epoxy resin shielding shell, thereby reducing the potential friction-induced hysteresis associated with conventional tank-type pressure sensing structures. The moisture unit utilizes a polyimide hygroscopic coating and dual-purposes the strain-isolated temperature unit as the temperature reference, achieving effective temperature compensation while enhancing sensor integration. The fully non-metallic packaging further ensures intrinsic insulation safety in high-voltage electromagnetic environments.

Experimental results demonstrate that the proposed sensor exhibits a temperature sensitivity of 17.1 pm/°C, a pressure sensitivity of approximately 4 nm/MPa, and a moisture sensitivity of 7.775 × 10^−4^ nm/%RS in transformer oil, along with excellent linearity, repeatability, and immunity to cross-sensitivity. These findings validate the effectiveness of the packaging architecture and compensation strategy. The integrated FBG sensor operates stably under strong electromagnetic interference and high-temperature oil immersion conditions, offering a high-sensitivity and high-reliability solution for online monitoring of non-electrical parameters in power transformers.

To further verify the engineering applicability of the proposed sensor, future work can be carried out in two directions. On the one hand, multi-point networking technology can be explored to enable distributed monitoring inside power transformers. On the other hand, accelerated aging tests and transient response characterization should be conducted to systematically evaluate the influence of long-term material operation on sensor performance. These studies will provide a basis for quantifying the engineering maturity of the sensor and identifying necessary design improvements. Ultimately, through integration with data acquisition and intelligent analysis systems, the proposed sensor is expected to be applied to transformer condition assessment and fault warning, promoting the evolution of power transformer monitoring toward a multi-parameter, integrated, and intelligent paradigm.

## Figures and Tables

**Table 1:** Specifications of the optical fibers.

Parameter	FBG Pressure	FBG Temperature	FBG Humidity
Fiber type	Single-mode silica fiber	Single-mode silica fiber	Single-mode silica fiber
Grating type	Uniform	Uniform	Uniform
Grating length	5 mm	5 mm	5 mm
Nominal central wavelength	1540.0 nm	1550.0 nm	1560.0 nm
Reflectivity	>90%	>90%	>90%
Side-mode suppression ratio	>20 dB	>20 dB	>20 dB

**Table 2:** Specifications of the FBG Interrogator.

Parameter	Specification	Parameter	Specification
Wavelength Range	1527–1568 nm	Interrogator Repeatability	±0.3 pm
Scanning Speed	2000 Hz	Interrogator Accuracy	±1 pm
Spectral sampling interval	40 pm	Communication Interface	RJ45

## Data Availability

Data will be made available upon request.
